# Vitamin D status and risk of rheumatoid arthritis: systematic review and meta-analysis

**DOI:** 10.1186/s41927-023-00325-y

**Published:** 2023-03-15

**Authors:** Joanna L. Clasen, Rachel Cole, Dagfinn Aune, Edward Sellon, Alicia K. Heath

**Affiliations:** 1 Department of Epidemiology and Biostatistics, School of Public Health, Imperial College London, Norfolk Place, London, W2 1PG UK; 2 Department of Nutrition, Oslo New University College, Oslo, Norway; 3 Department of Endocrinology, Morbid Obesity and Preventive Medicine, Oslo University Hospital, Oslo, Norway; 4 Oxford University Hospitals NHS Foundation Trust, Oxford, UK

**Keywords:** Vitamin D, 25-hydroxyvitamin D, Rheumatoid arthritis, Autoimmune disease

## Abstract

**Background:**

Vitamin D is important for immunomodulation and may play a role in autoimmune diseases. Studies have reported a high prevalence of vitamin D deficiency in rheumatoid arthritis (RA) patients, and vitamin D status, assessed by circulating 25-hydroxyvitamin D [25(OH)D] concentration, is inversely associated with RA disease activity. However, it is unclear whether vitamin D deficiency increases the risk of later developing RA. We conducted a systematic review and meta-analysis of pre-diagnostic 25(OH)D concentrations and risk of RA.

**Methods:**

Medline and Embase databases were searched in December 2021 using various keywords for ‘vitamin D’, ‘rheumatoid arthritis’, and ‘prospective study’. Publications identified from the search were screened for eligibility, studies were excluded if vitamin D status was measured at or after RA diagnosis, and data were extracted from relevant articles. Bayesian meta-analysis was used to estimate the summary relative risk (RR) and 95% credible interval (CrI) for risk of RA in relation to circulating 25(OH)D concentrations, as well as the between-study heterogeneity.

**Results:**

The search strategy yielded 908 records, of which 4 publications reporting on 7 studies, involving a total of 15,604 participants and 1049 incident RA cases, were included in the meta-analysis. There was no suggestion of an association between 25(OH)D concentration and subsequent risk of RA. The pooled RR per 25 nmol/L increment in 25(OH)D was 0.96 (95% CrI 0.82–1.13; *I*^2^ = 52%). No associations were evident in men (RR = 1.02, 95% CrI 0.65–1.61; *I*^2^ = 77%, 2 studies) or women (RR = 0.94, 95% CrI 0.73–1.22; *I*^2^ = 71%, 4 studies).

**Conclusions:**

This systematic review and meta-analysis did not identify evidence of an association between 25(OH)D and RA risk, but there was notable between-study heterogeneity and a lack of precision. Investigations in large-scale prospective studies with long follow-up or suitably designed Mendelian randomisation studies with consideration of potential non-linear relationships are needed to determine whether vitamin D is involved in RA aetiology.

**Supplementary Information:**

The online version contains supplementary material available at 10.1186/s41927-023-00325-y.

## Background

Rheumatoid arthritis (RA) is a progressive autoimmune disease affecting around 0.5–1% of adults globally [1, 2]. RA manifests with chronic inflammation of synovial joints due to hyperplasia of synovial membranes, with infiltration by innate and adaptive immune cells and pro-inflammatory cytokines [2, 3]. The inflamed synovium, when left untreated, damages nearby cartilage and juxta-articular bone, causing irreversible joint destruction [2, 3]. Patients with RA typically experience bilateral swelling and tenderness of the hands and feet, accompanied by joint stiffness, and can also have extra-articular symptoms and complications [2–4]. Untreated, RA can substantially impact daily activities and reduce quality of life, particularly among the working age population [2, 3]. The aetiology of RA is incompletely elucidated, but women are two to three times more likely than men to develop the disease, and it usually begins between ages 30 and 50 years although it can occur at any age [3, 4]. Other risk factors include genetic susceptibility (e.g. human leukocyte antigen (HLA)-DRB1), tobacco smoking, and obesity [2, 3, 5].

Vitamin D deficiency has been suggested to be associated with various immune-mediated inflammatory diseases including RA [6–13]. Immunomodulatory effects of vitamin D are mediated in part by acting upon antigen-presenting cells such as dendritic cells [12, 14]. The active form of vitamin D, 1,25-dihydroxyvitamin D, suppresses autoimmunity by inhibiting production of pro-inflammatory cytokines by T helper (Th) cells such as Th1 and Th17, inducing production of anti-inflammatory cytokines by Th2 cells, and activating T-regulatory cells (Tregs), thereby engendering a more tolerogenic rather than pro-inflammatory state [6, 9, 12, 14].

Several studies have shown that vitamin D deficiency is more prevalent, and 25-hydroxyvitamin D [25(OH)D] concentrations (which provide a measure of vitamin D status) are lower in RA patients than healthy controls [15, 16]. In addition, studies have reported that higher 25(OH)D concentrations are correlated with lower disease activity [8, 15–17], and vitamin D supplementation has been shown to improve some measures of disease activity in RA patients [18]. However, vitamin D deficiency in RA patients could be a consequence of the disease rather than a cause [19], and it is unknown whether suboptimal vitamin D status increases the risk of developing RA. Vitamin D is mainly obtained via endogenous synthesis in the skin during sun exposure, and dietary sources are minimal. Higher vitamin D intake has been reported to be associated with lower risk of RA [17], but the relationship between vitamin D status and RA is unclear. We therefore conducted a systematic review and meta-analysis to summarise published evidence on the association between circulating 25(OH)D concentrations and risk of incident RA.

## Methods

### Search strategy

An electronic literature search was performed in Medline and Embase databases to identify all eligible studies on the association between pre-diagnostic 25(OH)D concentrations and RA risk that were published from inception to 21 December 2021. The search strategy included keywords and MeSH terms covering a comprehensive list of possible variations of ‘vitamin D’ and its forms, ‘rheumatoid arthritis’, and prospective study designs. The full search strategy for Medline and Embase is listed in Additional File 1. The systematic review protocol was registered with Prospero on 2 August 2021 (CRD42021262855) [20].

### Study selection

Results from the database searches were merged and duplicates removed using Covidence, a web-based software platform for systematic review management [21]. Records were independently screened by two investigators (JLC and RC) based on titles and abstracts in the first step. The full texts of the remaining records were then independently screened for eligibility in duplicate and uncertainties regarding inclusion were resolved by discussion with all investigators. The reference lists of included papers were manually searched and a forward citation search was performed to ensure no potentially relevant studies were missed. The inclusion criteria were as follows: prospective cohort studies, case-cohort studies, nested case–control studies, or case–control studies with retrospective collection of exposure data which reported adjusted relative risk (RR) estimates (RRs, hazard ratios (HRs), odds ratios (ORs), incidence rate ratios) and 95% confidence intervals (CIs) for RA risk in relation to 25(OH)D; with 25(OH)D concentrations measured in blood samples collected prior to diagnosis of RA; and participants aged 18 years and older. The outcome of interest was RA diagnosis, by self-report or clinician report, using American College of Rheumatology (ACR), European Alliance of Associations for Rheumatology (formerly European League Against Rheumatism, EULAR) [22], or International Classification of Diseases (ICD) criteria. Conference abstracts were excluded because they are unlikely to contain all the information required for statistical analyses and for evaluation of study quality. If there were multiple publications from the same study population, only the most recent was included. Reviews, letters, comments, editorials, meta-analyses, meta-syntheses, prediction models, protocols, cross-sectional studies, case reports, and epidemiological studies in patient populations were excluded. We also manually excluded non-human studies and non-English publications.

### Data extraction

Data extracted from the eligible papers included: first author name, year of publication, country/location, study name or description, study period, duration of follow-up, number of participants or controls, number of cases, age and sex distribution, study design, outcome assessment, method of 25(OH)D measurement, mean/median 25(OH)D, comparison (the contrast or metric of 25(OH)D), RR/OR/HR and 95% CI, and any adjustments made for covariates. If several multivariable models were reported, the most fully adjusted effect estimate was extracted and used for the meta-analysis. If an article reported pooled results from more than one study, only the individual results were used in this meta-analysis. Data were extracted by one investigator (RC) and separately verified by two investigators (JLC and AKH).

### Study quality assessment

The quality of each of the studies included in the meta-analysis was assessed with the Newcastle–Ottawa scale [23]. Each study was appraised using the categories of selection of cases and controls, comparability, and ascertainment of the exposure for case–control studies, and participant selection, comparability, and ascertainment of the outcome of interest for cohort and nested studies, with a maximum possible score of 9 stars. For publications reporting on multiple studies, each study was rated separately.

### Statistical analyses

Prior to meta-analysis, results from all studies were converted to estimates for a 25 nmol/L increment in 25(OH)D. If a study reported results for 25(OH)D in ng/ml, concentrations were first converted to nmol/L by multiplying by 2.496. Given the low prevalence of RA, different effect measures (HRs, ORs) were considered equivalent to RRs and used directly. If a study reported results separately by sex, or other subgroups, but not overall, the subgroup-specific estimates were pooled using a fixed-effect model before inclusion in the meta-analysis. If a study reported a CI with insufficient precision (due to rounding), the CI was estimated using the reported p-value. Because few studies met the eligibility criteria for inclusion, we used a Bayesian meta-analysis approach to estimate the summary RR and 95% credible interval (CrI) for RA in relation to a 25 nmol/L increment in 25(OH)D concentration. The CrI, similar to a frequentist CI, quantifies the uncertainty around the point estimate. Heterogeneity of RRs across studies is to be expected, given the differences in populations, study designs, and methods for ascertainment of outcomes. A frequentist random-effects model can reliably handle heterogeneity in a meta-analysis of a large number of studies, however, these models are not well-suited to meta-analyses of few studies. A Bayesian approach to meta-analysis accounts for uncertainty in the estimate of heterogeneity inherent in such situations [24]. Additionally, a prior distribution for τ can be used to stabilize the heterogeneity estimate around a reasonable value. Empirically derived priors for τ, based on previously published meta-analyses, have been developed, and here we employed the prior based on studies examining non-pharmacological exposures and onset of a new chronic disease [25]. We reported I^2^, the percent of total variation that is due to true between-study heterogeneity, which is derived from the posterior distribution of τ. The prior distribution for the log RR is weakly informative (normal with mean 0 and standard deviation 0.56), based on the assumption that the RR will have a magnitude of less than 3.

To investigate potential heterogeneity in the results, subgroup analyses were conducted by sex among studies that reported sex-specific estimates. To evaluate whether the results were driven by one large study or a study with an extreme result, a sensitivity analysis was performed sequentially excluding each study from the analysis one at a time and assessing the impact on the summary estimate. As a further sensitivity analysis, frequentist random-effects models were run to estimate the summary RR and 95% CI for risk of RA per 25 nmol/L increment in 25(OH)D. Risk of publication bias was evaluated by visually inspecting funnel plots and using the Egger linear regression test [26] and Begg rank correlation test [27].

All statistical analyses were performed using R version 4.1.0 [28], and the metafor version 3.0–2 [29] and bayesmeta version 2.7 [30] packages.

## Results

### Study selection

**Fig. 1:**
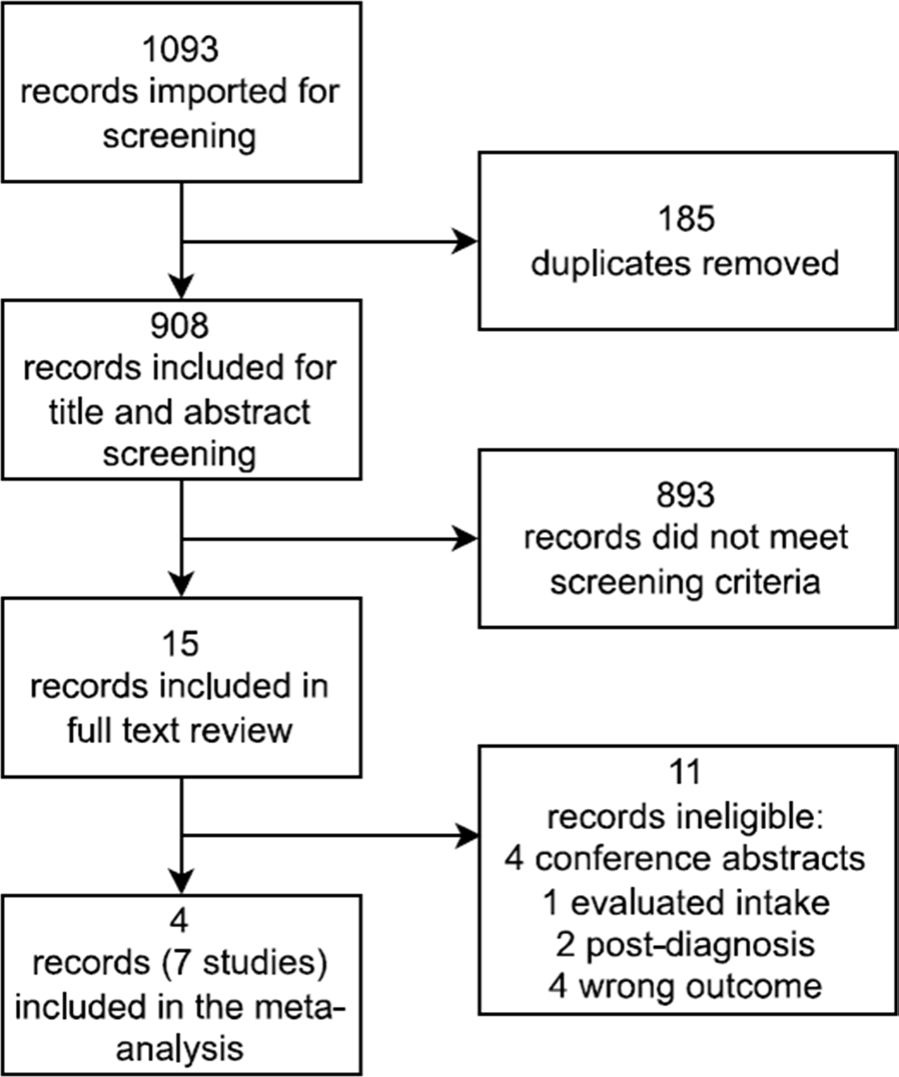
Flow diagram of study selection for the meta-analysis of the association between 25-hydroxyvitamin D concentration and risk of rheumatoid arthritis

The flow diagram of study selection is shown in Fig. 1. A total of 1093 records (221 from Medline and 872 from Embase) were identified from the search strategy. After removing 185 duplicates, 908 records remained for screening. Based on the titles and abstracts, 893 records were deemed ineligible, leaving 15 potentially relevant records to be evaluated for eligibility by assessing their full texts. Of these 15 records, 4 were conference abstracts, 1 evaluated vitamin D intake, 2 measured circulating 25(OH)D after diagnosis, and 4 did not evaluate RA as an outcome, and were therefore excluded. Four articles including a total of seven studies were included in the meta-analysis of 25(OH)D concentrations and risk of RA.

### Study characteristics

The characteristics of included studies are summarised in Table 1. All papers were published between 2014 and 2018, and one included data from two studies [31] and one from three studies [32]. Among the total of seven included studies, three (two publications) were conducted in the USA [31, 33], three (one publication) in Denmark [32], and one in Sweden [34]. Three of the studies were nested case–control studies [31, 34], one was a case–control study with retrospective 25(OH)D measurements [33], and three were prospective cohort studies [32]. The number of RA cases in individual studies ranged from 8 in the Health2006 study [32] to 515 in the Northern Sweden Health and Disease Study [34]. Five studies (three publications) included both men and women [32–34], and two studies (one publication) included women only [31]. Two of the studies that included both men and women reported sex-specific results [33, 34], and only one of these studies [33] additionally reported an overall estimate. Only three studies (two publications) [31, 33] reported effect estimates for 25(OH)D categories, whereas all studies presented results for 25(OH)D modelled continuously. All studies adjusted for potential confounders including age, sex, smoking, and body mass index.

**Table 1:** Characteristics of studies included in the meta-analysis of 25-hydroxyvitamin D concentration and risk of rheumatoid arthritis

Author, year of publication	Study name or description; location	Study period; follow-up duration	Number of participants and cases; sex; age	Study design; outcome assessment	Method of 25(OH)D measurement	25(OH)D comparison	RR, OR, or HR and 95% CI	Adjustments for covariates	NOS score
Brink et al. 2018 [34]	Northern Sweden Health and Disease Study (NSHDS), comprising the Västerbotten intervention program [VIP], monitoring trends and determinants in cardiovascular disease [MONICA] and the Mammography screening project Sweden	mean 6.2 years to diagnosis	515 cases, 267 controls Women: 365 cases, 188 controls Men: 150 cases, 79 controls Mean age: 52.2 years for cases, 53.3 years for controls	Nested case–control study Case criteria: patients fulfilling the 1987 American Rheumatism Association classification criteria for RA, with blood sampled before date of symptom onset Controls randomly selected from the same cohorts, matched 2:1 for age, sex, date of blood sample	Liquid chromatography-tandem mass spectrometry	Per 1 nmol/L^a^	OR Women 0.99 (0.99–1.00) Men 1.00 (0.99–1.02)	Age, season, BMI, ever smoking, educational level	8
					Mean 25(OH)D Cases: 53.8 nmol/L Controls: 54.5 nmol/L				
Cote et al. 2014 [33]	Electronic health records in the Geisinger Health System (GHS) USA	2001–2012 median 125.5 days (range 1–2247 days) between 25(OH)D measurement and incident RA	270 cases, 1341 controls Women: 225 cases, 1125 controls Men: 45 cases, 216 controls Median age: 62.4 years at diagnosis	Case–control study with retrospective 25(OH)D measurements Case criteria: incident RA defined as ICD-9 code 714.0 twice by a rheumatologist, with at least one 25(OH)D level recorded prior to diagnosis Controls selected from the general GHS population, matched 5:1 for age and gender	DiaSorin radioimmunoassay (2001–September 2006); liquid chromatography-tandem mass spectrometry (October 2006–May 2009); DiaSorin LIAISON (June 2009–November 2012) median 25(OH)D 31 ng/ml (IQR 23–39)	Continuous, per 1 ng/ml^b^	OR 0.996 (0.987–1.005) Women 0.995 (0.985–1.006) Men 0.997 (0.977–1.018)	Age and sex as matching factors and adjusted for BMI and smoking status (ever/never)	6
						< 30 vs ≥ 30 ng/ml	0.982 (0.748–1.291) Women 0.929 (0.685–1.259) Men 1.226 (0.647–2.324)		
						< 20 vs ≥ 20 ng/ml	1.124 (0.803–1.573) Women 1.198 (0.829–1.730) Men 0.855 (0.361–2.025)		
Hiraki et al. 2014 [31]	Nurses’ Health Study (NHS) USA	Recruited 1976, blood sampling 1989–1990, follow-up until June 2006 Mean 7.8 years between blood draw and RA diagnosis	120 cases, 357 controls Women only Mean age 56.0 years at blood draw; 63.8 years at RA diagnosis	Nested case–control study Case criteria: self-reported on biennial follow-up survey, followed by CTD Screening Questionnaire. Confirmed against standardized ACR classification criteria for RA through review of medical records by two board-certified rheumatologists Controls selected from participants with stored blood, matched 3:1 on age (± 1 year), menopausal status and postmenopausal hormone use, month and year of blood collection, time of day and fasting status at blood draw	Radioimmunoassay Mean 25(OH)D not reported	Continuous, per 1 ng/ml increase	OR^c^ 1.01 (0.99–1.04)	Smoking status (ever/never), parity and breastfeeding, alcohol consumption, BMI, median income, region of USA residence; conditioned on matching factors (age, date of blood draw, fasting status, menopausal status, hormone use)	7
						> 20 vs ≤ 20 ng/ml	1.30 (0.78–2.16)		
						Highest vs lowest quartile	1.51 (0.75–3.05)		
						Per 1 ng/ml increase	Stratified by time between blood draw and diagnosis: 3 months– < 4 years 0.94 (0.85–1.04) ≥ 4 years 1.02 (0.99–1.05)		
Hiraki et al. 2014 [31]	Nurses’ Health Study II (NHSII) USA	Recruited 1989, blood sampling 1996–1999, follow-up until 2007 Mean 4.2 years between blood draw and RA diagnosis	46 cases, 133 controls Women only Mean age 44.5 years at blood draw; 48.5 at RA diagnosis	Nested case–control study Case criteria: self-reported on biennial follow-up survey, followed by CTD Screening Questionnaire. Confirmed against standardized ACR classification criteria for RA through review of medical records by two board-certified rheumatologists Controls selected from participants with stored blood, matched 3:1 on age (± 1 year), menopausal status and postmenopausal hormone use, month and year of blood collection, time of day and fasting status at blood draw, and additionally matched to premenopausal cases based on menstrual cycle phase	Radioimmunoassay Mean 25(OH)D not reported	Continuous, per 1 ng/ml increase	OR^c^ 0.98 (0.94–1.03)	Smoking status (ever/never), parity and breastfeeding, alcohol consumption, BMI, median income, region of USA residence; conditioned on matching factors (age, date of blood draw, fasting status, menopausal status, hormone use, and menstrual cycle timing)	7
						> 20 vs ≤ 20 ng/ml	0.97 (0.38–2.50)		
						Highest vs lowest quartile	0.66 (0.17–2.56)		
						Per 1 ng/ml increase	Stratified by time between blood draw and diagnosis: 3 months ≤ 4 years 0.80 (0.64–0.99) ≥ 4 years 1.00 (0.92–1.08)		
Skaaby et al 2015 [32]	Monica10 Denmark	Recruited 1993–1994, follow-up until 31 Dec 2010 Median 16.4 years	Total 2649; 43 cases 50.2% men Mean age 55.4 years (range 40–71 years)	Population-based study, random sample of the population recruited from the Danish Central Personal Register Seropositive RA (ICD-8 codes 712.19, 712.39, 712.59 or ICD-10 codes M05-M06) identified by linkage to the Danish National Patient Register which contains information on admissions to Danish hospitals since 1978	IDS-SYS 25-Hydroxy Vitamin D method Mean 25(OH)D 64.7 nmol/L	Per 10 nmol/L higher 25(OH)D	HR^d^ 1.01 (0.88–1.15)	Age, sex, education, season of blood sample, physical activity, smoking habits, alcohol intake, BMI, systolic and diastolic blood pressure, serum total cholesterol, serum triglycerides	9
Skaaby et al 2015 [32]	Inter99 Denmark	Recruited 1999–2001, follow-up until 31 Dec 2010 Median 11.0 years	Total 6497; 47 cases 49.2% men Mean age 46.1 years (range 30–60 years)	Population-based study, random sample of the population recruited from the Danish Central Personal Register, randomised controlled trial that investigated effects of lifestyle interventions on cardiovascular disease Seropositive RA (ICD-8 codes 712.19, 712.39, 712.59 or ICD-10 codes M05-M06) identified by linkage to the Danish National Patient Register which contains information on admissions to Danish hospitals since 1978	High-performance liquid chromatography Mean 25(OH)D 51.2 nmol/L	Per 10 nmol/L higher 25(OH)D	HR^d^ 0.94 (0.82–1.08)	Age, sex, education, season of blood sample, physical activity, smoking habits, alcohol intake, BMI, systolic and diastolic blood pressure, serum total cholesterol, serum triglycerides	9
Skaaby et al. 2015 [32]	Health2006 Denmark	Recruited 2006–2008, follow-up until 31 Dec 2010 Median 3.5 years	Total 3409; 8 cases 44.9% men Mean age 49.4 years (range 18–69 years)	Population-based study, random sample of the population recruited from the Danish Central Personal Register Seropositive RA (ICD-8 codes 712.19, 712.39, 712.59 or ICD-10 codes M05-M06) identified by linkage to the Danish National Patient Register which contains information on admissions to Danish hospitals since 1978	Roche Diagnostics immunoassay using Cobas e411 Mean 25(OH)D 44.3 nmol/L	Per 10 nmol/L higher 25(OH)D	HR^d^ 1.02 (0.63–1.67)	Age, sex, education, season of blood sample, physical activity, smoking habits, alcohol intake, BMI, systolic and diastolic blood pressure, serum total cholesterol, serum triglycerides	8

*ACR* American College of Rheumatology, *BMI* body mass index, *CI* confidence interval, *CTD* connective tissue disease, *HR* hazard ratio, *ICD* International Classification of Diseases, *IQR* interquartile range, *NOS* Newcastle–Ottawa scale, *OR* odds ratio, *RA* rheumatoid arthritis, *RR* relative risk, 25(OH)D 25-hydroxyvitamin D

^a^The 25(OH)D metric was not reported but was assumed to be per 1 nmol/L based on the information presented in the publication

^b^The 25(OH)D metric was not reported but was assumed to be per 1 ng/ml based on the information presented in the publication

^c^ORs for NHS and NHSII combined were 1.00 (95% CI 0.98–1.03) per 1 ng/ml increase, 1.21 (95% CI 0.77–1.90) for > 20 versus ≤ 20 ng/ml, and 1.26 (95% CI 0.68–2.36) for highest versus lowest quartile of 25(OH)D. Combined ORs per 1 ng/ml increase in 25(OH)D were 0.91 (95% CI 0.83–1.00) for 3 months to < 4 years, and 1.02 (95% CI 0.99–1.05) for ≥ 4 years before RA diagnosis

^d^Pooled HR for Monica10, Inter99 and Health 2006 combined was 0.97 (95% CI 0.89–1.07) per 10 nmol/L higher 25(OH)D

### Quality assessment

The average study quality score using the Newcastle–Ottawa scale was 7.7 out of 9 (Table 1). Differences in representativeness of the cases or cohort, laboratory methods used for biochemical analyses, and assessment of the outcome accounted for most of the variability in quality across studies.

### Meta-analysis of 25(OH)D concentration and risk of RA

The seven studies included in the linear dose–response meta-analysis comprised a total of 15,604 participants and 1049 RA cases. Brink et al. [34] did not provide an overall risk estimate, but separate results for men and women, which we combined using a fixed-effect model, yielding a RR of 0.94 (95% CI 0.79–1.12) per 25 nmol/L increment in 25(OH)D; this estimate was subsequently used in the main meta-analysis.

**Fig. 2:**
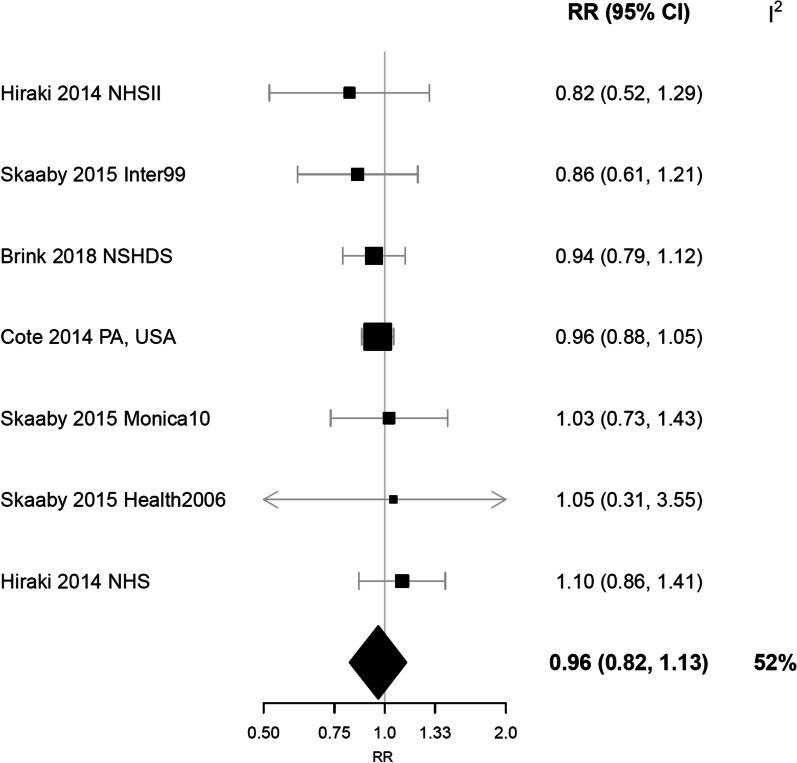
Linear dose–response meta-analysis of 25-hydroxyvitamin D concentration and risk of rheumatoid arthritis. Estimates are for a 25 nmol/L increment in 25(OH)D. Study-specific RRs are plotted as squares, with the area of each square inversely proportional to the variance of the logRR, and corresponding CIs are plotted as horizonal lines. The diamond represents the pooled RR and 95% credible interval. CI, confidence interval; 25(OH)D, 25-hydroxyvitamin D; NHS, Nurses’ Health Study; NSHDS, Northern Sweden Health and Disease Study; RR, relative risk

**Fig. 3:**
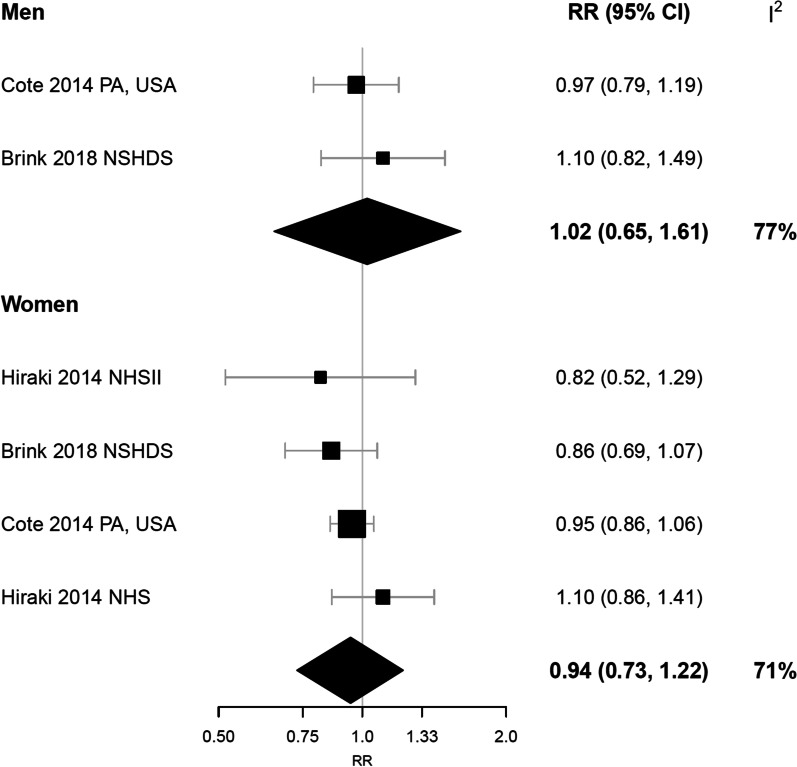
Linear dose–response meta-analysis of 25-hydroxyvitamin D concentration and risk of rheumatoid arthritis in men and women. Estimates are for a 25 nmol/L increment in 25(OH)D. Study-specific RRs are plotted as squares, with the area of each square inversely proportional to the variance of the logRR, and corresponding CIs are plotted as horizonal lines. The diamond represents the pooled RR and 95% credible interval. CI, confidence interval; 25(OH)D, 25-hydroxyvitamin D; NHS, Nurses’ Health Study; NSHDS, Northern Sweden Health and Disease Study; RR, relative risk

The summary RR per 25 nmol/L increment in 25(OH)D was 0.96 (95% CrI 0.82–1.13) and there was moderate heterogeneity between studies (I^2^ = 52%) (Fig. 2). No differences were observed by sex, with no association in either men (RR per 25 nmol/L increment in 25(OH)D = 1.02, 95% CrI 0.65–1.61; I^2^ = 77%) or women (RR = 0.94, 95% CrI 0.73–1.22; I^2^ = 71%) (Fig. 3). Similar summary estimates were obtained using frequentist random-effects models (overall RR = 0.96, 95% CI 0.90–1.04; men RR = 1.01, 95% CI 0.85–1.20; women RR = 0.95, 95% CI 0.87–1.04), with no indication of between-study heterogeneity (I^2^ = 0%) (Additional File 1: Fig. S1).

In sensitivity analyses sequentially excluding each study from the meta-analysis one at a time, there was little change in the results. The summary RR per 25 nmol/L increment in 25(OH)D ranged from 0.94 (95% CrI 0.77–1.13) when the Nurses’ Health Study (NHS) [31] was omitted, to 0.98 when omitting either the Inter99 study [32] (95% CrI 0.81–1.18) or NHSII [31] (95% CrI 0.82–1.17) (Additional File 1: Fig. S2).

Visual inspection of the funnel plot (Additional File 1: Fig. S3) and Egger’s (*P* = 0.95) and Begg’s (*P* = 0.56) tests did not suggest publication bias, but the low number of studies precluded reliable assessment.

## Discussion

This systematic review revealed a scarcity of studies investigating vitamin D status in relation to risk of developing RA. The meta-analysis did not find evidence of an association between 25(OH)D concentration and risk of RA, but the number of studies was small, most studies contained few cases, and estimates were imprecise. Using a Bayesian approach, moderate to high between-study heterogeneity was identified.

There is inconsistent evidence for a role of vitamin D in RA development. In agreement with our findings, a Mendelian randomisation analysis did not find evidence of a causal association between 25(OH)D and risk of RA [35]. Another study, which was not included in this review due to inadequate data, similarly reported that vitamin D deficiency was not associated with risk of RA [36]. However, these results are inconsistent with a meta-analysis of three prospective cohort studies which found an inverse association between vitamin D intake and risk of RA and suggested a possible beneficial role of vitamin D [17]. Nevertheless, vitamin D intake is not directly comparable to vitamin D status, which was the focus of our review.

Although this meta-analysis did not find any association between vitamin D status and RA risk, it is possible that vitamin D plays a role in disease severity and progression. Several studies have reported favourable associations between higher 25(OH)D concentrations or vitamin D supplementation and lower disease activity in patients with RA [15–18]. In addition, meta-analyses have demonstrated that RA patients tend to have lower 25(OH)D concentrations than healthy controls [15, 16]. However, systemic inflammation in RA patients with high disease activity, and certain lifestyle behaviours such as less outdoor activity and sun exposure and lower dietary intake of vitamin D in those with more severe disease, might contribute to lower 25(OH)D concentrations [19]. The direction of the mechanistic link between vitamin D status and RA, if it does exist, is therefore unclear.

This systematic review focused on the relationship between pre-diagnostic 25(OH)D concentration and subsequent risk of RA, and thus the findings cannot be directly compared with studies assessing the post-diagnostic association in patient populations. The duration of follow-up (average time between 25(OH)D measurement and diagnosis) in some of the individual studies was only a few years and it is possible that some participants might have had underlying undiagnosed disease when blood samples were collected. An inverse association between 25(OH)D and RA risk was reported among women in NHSII with 25(OH)D measured less than 4 years before symptom onset (OR = 0.80, 95% CI 0.64–0.99 per 1 ng/ml (2.5 nmol/L) increase in 25(OH)D), but not for 25(OH)D measurements 4 or more years prior to diagnosis [31]. Subclinical inflammatory processes might reduce 25(OH)D prior to diagnosis [19]; patients are often not diagnosed with RA until months or even years after initial symptom onset, by which stage synovitis would have been present for some time [2]. Because the number of studies was small, we were unable to evaluate associations according to the length of time between 25(OH)D assessment and RA diagnosis. Further, individual studies generally lack repeat 25(OH)D measurements and therefore have not examined long-term vitamin D status, or changes in relation to RA. It is possible that chronic vitamin D deficiency or low 25(OH)D concentrations during certain periods of life might play a role in RA aetiology, and further studies are needed to investigate this possibility.

This systematic review identified very few prospective studies that have investigated the relationship between pre-diagnostic 25(OH)D and risk of RA. The included studies had few RA cases and participants were predominantly women. Moreover, there was a lack of geographical and ethnic diversity, with results available from studies in only three countries (USA, Denmark, and Sweden), and almost all participants were of European descent. Further research is needed in studies with large numbers of participants that include those of different ethnicities, different geographical locations, broader age groups, and adequate numbers of cases.

A limitation of this meta-analysis was the wide variability in assays used to measure 25(OH)D in the individual studies. Measurements of 25(OH)D exhibit considerable inter-assay and inter-laboratory differences, and there is a lack of standardisation of methods, which hinders the ability to directly compare results from studies that report results based on unstandardised 25(OH)D measurements [37, 38]. We examined 25(OH)D modelled continuously instead of according to clinical categories or specific cut-offs, which limited the possibility of the meta-analysis being affected by misclassification due to assay variability and lack of standardisation of 25(OH)D measurements.

Emerging evidence suggests that associations of 25(OH)D with various health outcomes appear to be non-linear, and if vitamin D is beneficial, it is likely to be relevant only for those with vitamin D deficiency [39, 40]. However, in this meta-analysis it was not possible to investigate the non-linear dose–response relationship with RA because insufficient studies reported results for 25(OH)D categories. Moreover, the range of 25(OH)D among the studies’ participants did not cover the potential wide spectrum of 25(OH)D concentrations that truly exists in the general population, so we were unable to examine the relationship between 25(OH)D and RA at low 25(OH)D concentrations and cannot rule out the possibility of such an association. In the study by Brink et al. [34] the mean 25(OH)D concentration was 54.5 nmol/L in controls and 53.8 nmol/L in cases (levels which are considered to be adequate, i.e. > 50 nmol/L). Similarly, mean 25(OH)D concentrations were 64.7 nmol/L in Monica10, 51.2 nmol/L in Inter99, and 44.3 nmol/L in Health2006 [32]. Further, in the study by Cote et al. [33], only 18% of participants were classified in the ‘vitamin D deficient’ category. Thus, overall the included studies mainly comprised vitamin D replete individuals and this limited the ability of this meta-analysis to assess whether vitamin D deficiency is associated with risk of RA. Elucidating this potential relationship will require additional large-scale prospective studies involving participants with a greater range of 25(OH)D concentrations. Stratified/non-linear Mendelian randomisation analyses are also warranted to evaluate a possible non-linear relationship of 25(OH)D with RA risk.

A strength of this meta-analysis was the use of a Bayesian approach, which allowed for a more robust estimation of the between-study heterogeneity compared with frequentist methods, which is particularly useful when there are few studies included.

## Conclusions

In summary, this systematic review and meta-analysis found no association between vitamin D status and risk of RA. Nevertheless, few studies have investigated this relationship, and no firm conclusions can be drawn in the context of limited data. Further large-scale prospective studies with long follow-up durations, a larger number of cases, and enriched with participants with a broad range of 25(OH)D (particularly those with very low concentrations) are warranted to determine whether vitamin D deficiency is associated with the risk of developing RA.

## Supplementary Information

**Additional file 1**. Medline search strategy. Embase search strategy.** Figure S1**. Frequentist random-effects meta-analysis of 25-hydroxyvitamin D concentration and risk of rheumatoid arthritis.** Figure S2**. Linear dose-response meta-analysis of 25-hydroxyvitamin D concentration and risk of rheumatoid arthritis, omitting each individual study one at a time.** Figure S3**. Funnel plot for the meta-analysis of 25-hydroxyvitamin D concentration and risk of rheumatoid arthritis.

## Data Availability

All data analysed during this study are reported within this article or in the cited references.

## Abbreviations

CI: Confidence interval
CrI: Credible interval
HR: Hazard ratio
25(OH)D: 25-Hydroxyvitamin D
NHS: Nurses’ Health Study
OR: Odds ratio
RA: Rheumatoid arthritis
RR: Relative risk
