# Supplementary material for: Vitamin D status and risk of rheumatoid arthritis: systematic review and meta-analysis
Source: BMC Rheumatol. 2023 Mar 15;7:3. doi: 10.1186/s41927-023-00325-y (PMC10015722; doi:10.1186/s41927-023-00325-y)
Supplement: Supplementary file 1 — Additional file 1. Medline search strategy. Embase search strategy. Figure S1. Frequentist random-effects meta-analysis of 25-hydroxyvitamin D concentration and risk of rheumatoid arthritis. Figure S2. Linear dose-response meta-analysis of 25-hydroxyvitamin D concentration and risk of rheumatoid arthritis, omitting each individual study one at a time. Figure S3. Funnel plot for the meta-analysis of 25-hydroxyvitamin D concentration and risk of rheumatoid arthritis. [file 41927_2023_325_MOESM1_ESM.docx]

**Vitamin D status and risk of rheumatoid arthritis: systematic review and meta-analysis**

Joanna L Clasen, Rachel Cole, Dagfinn Aune, Edward Sellon, Alicia K Heath

**Supplementary material**

**Medline search strategy:**

| 1. | Vitamin D Deficiency/ |
| --- | --- |
| 2. | Vitamin D/ |
| 3. | Cholecalciferol/ |
| 4. | Ergocalciferols/ |
| 5. | Calcifediol/ |
| 6. | vitamin D.ab,ti. |
| 7. | 25-hydroxyvitamin D.ab,ti. |
| 8. | 25-hydroxy vitamin D.ab,ti. |
| 9. | "25(OH)D".ab,ti. |
| 10. | 25OHD.ab,ti. |
| 11. | 1 or 2 or 3 or 4 or 5 or 6 or 7 or 8 or 9 or 10 |
| 12. | Arthritis, Rheumatoid/ |
| 13. | rheumatoid arthritis.ab,ti. |
| 14. | 12 or 13 |
| 15. | Cohort Studies/ |
| 16. | Prospective Studies/ |
| 17. | follow-up.ab,ti. |
| 18. | cohort*.ab,ti. |
| 19. | case-control.ab,ti. |
| 20. | case-cohort.ab,ti. |
| 21. | prospective*.ab,ti. |
| 22. | retrospective*.ab,ti. |
| 23. | longitudinal.ab,ti. |
| 24. | observational.ab,ti. |
| 25. | survey*.ab,ti. |
| 26. | hazard ratio*.ab,ti. |
| 27. | relative risk*.ab,ti. |
| 28. | odds ratio*.ab,ti. |
| 29. | inciden*.ab,ti. |
| 30. | 15 or 16 or 17 or 18 or 19 or 20 or 21 or 22 or 23 or 24 or 25 or 26 or 27 or 28 or 29 |
| 31. | 11 and 14 and 30 |

**Embase search strategy:**

| 1. | vitamin D deficiency/ |
| --- | --- |
| 2. | vitamin D/ |
| 3. | colecalciferol/ |
| 4. | ergocalciferol/ |
| 5. | calcifediol/ |
| 6. | 25 hydroxyvitamin D/ |
| 7. | vitamin D.ab,ti. |
| 8. | 25 hydroxyvitamin D.ab,ti. |
| 9. | 25 hydroxy vitamin D.ab,ti. |
| 10. | "25(OH)D".ab,ti. |
| 11. | 25OHD.ab,ti. |
| 12. | 1 or 2 or 3 or 4 or 5 or 6 or 7 or 8 or 9 or 10 or 11 |
| 13. | rheumatoid arthritis/ |
| 14. | rheumatoid arthritis.ab,ti. |
| 15. | 13 or 14 |
| 16. | 12 and 15 |
| 17. | cohort analysis/ |
| 18. | prospective study/ |
| 19. | case control study/ |
| 20. | follow-up.ab,ti. |
| 21. | cohort*.ab,ti. |
| 22. | case-cohort.ab,ti. |
| 23. | case-control.ab,ti. |
| 24. | prospective*.ab,ti. |
| 25. | retrospective*.ab,ti. |
| 26. | longitudinal.ab,ti. |
| 27. | observational.ab,ti. |
| 28. | survey.ab,ti. |
| 29. | hazard ratio*.ab,ti. |
| 30. | relative risk*.ab,ti. |
| 31. | odds ratio*.ab,ti. |
| 32. | inciden*.ab,ti. |
| 33. | 17 or 18 or 19 or 20 or 21 or 22 or 23 or 24 or 25 or 26 or 27 or 28 or 29 or 30 or 31 or 32 |
| 34. | 16 and 33 |

**Supplementary Figure 1: Frequentist random-effects meta-analysis of 25-hydroxyvitamin D concentration and risk of rheumatoid arthritis.** Estimates are for a 25 nmol/L increment in 25(OH)D. Study-specific RRs are plotted as squares, with the area of each square inversely proportional to the variance of the logRR, and corresponding CIs are plotted as horizonal lines. The diamond represents the pooled RR and 95% CI. CI, confidence interval; 25(OH)D, 25-hydroxyvitamin D; NHS, Nurses’ Health Study; NSHDS, Northern Sweden Health and Disease Study; RR, relative risk.

**Supplementary Figure 2:** **Linear dose-response meta-analysis of 25-hydroxyvitamin D concentration and risk of rheumatoid arthritis, omitting each individual study one at a time.** Estimates are for a 25 nmol/L increment in 25(OH)D. Study-specific RRs are plotted as squares, with the area of each square inversely proportional to the variance of the logRR, and corresponding CIs are plotted as horizonal lines. The diamond represents the pooled RR and 95% credible interval. CI, confidence interval; NHS, Nurses’ Health Study; NSHDS, Northern Sweden Health and Disease Study; 25(OH)D, 25-hydroxyvitamin D; RR, relative risk.

**Supplementary Figure 3: Funnel plot for the meta-analysis of 25-hydroxyvitamin D concentration and risk of rheumatoid arthritis.**
